# The utility of a Bayesian predictive model to forecast neuroinvasive West Nile virus disease in the United States of America, 2022

**DOI:** 10.1371/journal.pone.0290873

**Published:** 2023-09-08

**Authors:** Maggie S. J. McCarter, Stella Self, Kyndall C. Dye-Braumuller, Christopher Lee, Huixuan Li, Melissa S. Nolan

**Affiliations:** 1 Department of Epidemiology and Biostatistics, University of South Carolina, Columbia, SC, United States of America; 2 Department of Computer Science and Engineering, University of South Carolina, Columbia, SC, United States of America; Clinton Health Access Initiative, UNITED STATES

## Abstract

Arboviruses (arthropod-borne-viruses) are an emerging global health threat that are rapidly spreading as climate change, international business transport, and landscape fragmentation impact local ecologies. Since its initial detection in 1999, West Nile virus has shifted from being a novel to an established arbovirus in the United States of America. Subsequently, more than 25,000 cases of West Nile neuro-invasive disease have been diagnosed, cementing West Nile virus as an arbovirus of public health importance. Given its novelty in the United States of America, high-risk ecologies are largely underdefined making targeted population-level public health interventions challenging. Using the Centers for Disease Control and Prevention ArboNET neuroinvasive West Nile virus data from 2000–2021, this study aimed to predict neuroinvasive West Nile virus human cases at the county level for the contiguous USA using a spatio-temporal Bayesian negative binomial regression model. The model includes environmental, climatic, and demographic factors, as well as the distribution of host species. An integrated nested Laplace approximation approach was used to fit our model. To assess model prediction accuracy, annual counts were withheld, forecasted, and compared to observed values. The validated models were then fit to the entire dataset for 2022 predictions. This proof-of-concept mathematical, geospatial modelling approach has proven utility for national health agencies seeking to allocate funding and other resources for local vector control agencies tackling West Nile virus and other notifiable arboviral agents.

## Introduction

West Nile virus (WNV) is an arthropod-borne virus (arbovirus) in the family Flaviviridae (genus *Flavivirus*) first isolated in the West Nile district of Uganda in 1937 [1]. Since its introduction into the Northern Hemisphere in 1999, it has become widely distributed in North and Central America and is most likely established in South America [2, 3]. It is thought that WNV is the most widely distributed arbovirus globally [4]. The primary mosquito vectors of WNV are *Culex* spp., specifically those in the *Culex pipiens* L. complex, *Culex tarsalis* Coquillett, *Culex restuans* Theobald, *Culex nigripalpus* Theobald, and the *Culex univittatus* Theobald complex [5, 6]. WNV is maintained in the environment through an enzootic cycle between these mosquito vectors and Passeriformes birds as vertebrate reservoirs and amplifying hosts during epidemics [7, 8]. The virus can be spread to additional vertebrates such as humans when a bridge vector mosquito species feeds on a mammal; however, humans do not produce high enough viremia to continue the spread of the virus into additional mosquitoes with subsequent bites [9, 10]. Clinical symptoms of disease typically appear in approximately 20% of those infected; these include fever, headache, body aches, joint pains, vomiting, diarrhea, or rash [11]. Severe illness affecting the central nervous system with encephalitis and meningitis can occur in <1% of cases, called West Nile neuroinvasive disease (WNND) [12]. In the continental United States of America (USA), WNV disease is the leading cause of mosquito-borne illness [13]. Cumulative estimates of its impact have suggested WNV has caused more than 25,000 neuroinvasive disease cases and 7 million infections since its introduction into the USA [14]. In addition to its public health impacts, WNV has also produced a heavy economic burden, where the cost of acute clinical care and subsequent long-term costs associated with infection is estimated at $56 million annually [15, 16].

With these estimated case and medical cost burdens, the USA must stay vigilant and active in mosquito and mosquito-borne disease surveillance. The National Association of County and City Health Officials (NACCHO) published a national Vector Control Assessment in 2020, finding that less than a quarter of mosquito and vector control programs in the USA are “fully capable” regarding mosquito surveillance and control capacity, in which such programs perform all 10 core and supplemental vector control capabilities, as outlined by NACCHO [17]. Additional regional and state vector control capacity surveys have reported similar results: the majority of mosquito and vector control agencies or programs are not prepared to handle major vector-borne disease outbreaks or are not proactive in various surveillance capabilities, leaving the USA vulnerable [18–20]. This vulnerability, in combination with anthropogenic climate change, urbanization, and international import and export, has set the stage for increased arboviral transmission in the USA [21, 22]. Clearly, empirical knowledge of disease cases and vector species infection is not enough to prevent disease. Vector-borne disease forecast modeling, relying on input from a multitude of disciplines including epidemiology, entomology, ecology, and biology to predict risk can be used to supplement empirical knowledge and further prevent continued spread and additional outbreaks.

Mathematical and computational modeling for epidemiology has significantly advanced in recent years and is commonly used for its insight into spatio-temporal transmission dynamics, synthesis of multi-disciplinary inputs, and overall reduction in cost compared to traditional surveillance [23]. Given the zoonotic nature of many vector-borne diseases, these modeling techniques are particularly useful when traditional disease surveillance cannot reach all potential animal reservoirs or hosts. Many approaches exist for forecasting WNV, including spatial and spatio-temporal regression models, compartmental models, and machine learning methods [24–29]. Bayesian regression models fit using Markov chain Monte Carlo (MCMC) methods have been heavily used for decades in disease prediction, and have shown to be effective for the prediction of WNV and corresponding associations [30–32]. However, MCMC approaches are often computationally expensive. The Integrated Nested Laplace Approximation (INLA) technique is a streamlined alternative to the traditional MCMC [33]. Contrary to the MCMC approach of estimating the joint posterior distribution of the parameters, the INLA model uses marginal inference on individual posteriors. This greatly reduces computation time while remaining precise in estimation.

In May 2022, the CDC released a call for that year’s WNND predictions to aid in more efficient allocation of resources and preparedness for this vector-borne disease. This paper describes a prediction model developed for this challenge to ultimately forecast WNND cases at the county level for the contiguous USA using a spatio-temporal Bayesian negative binomial regression, fit using an INLA approach. This is one of the first studies to predict human neuroinvasive WNV incidence for the contiguous USA at the county level and has direct public health applicability.

## Methods

### WNND data

Data on WNND were obtained from the Centers for Disease Control and Prevention’s ArboNET, a national arboviral surveillance database maintaining data on arboviral infections in humans, veterinary disease, and vector and host prevalence and health. ArboNET human cases are collected from clinicians who diagnose patients with an arboviral disease, and include both neuroinvasive and non-neuroinvasive WNV cases [34]. Only neuroinvasive cases, WNND, were included in this study.

### Covariates

Covariates were selected through a thorough literature search and input from experts in the field including medical entomologist coinvestigators and the CDC WNV forecasting team. As older populations are more susceptible to WNV infection, we included a proportion of population aged 65 years of age and older variable from each county, obtained from the US Census Bureau [35].

Avian host data for the three primary WNV amplifying host bird species was collected from the eBird database, a global citizen-science database dedicated to facilitating understanding of avian patterns [36]. Data was obtained for all sightings of American crows (*Corvus brachyrynchos*), blue jays (*Cyanocitta cristata*), and house sparrows (*Passer domesticus*) in the contiguous USA from January 1, 2000 to December 31, 2021, and linear extrapolation was used to estimate avian population for the year 2022. Available data included the latitude-longitude location of the sighting and the number of birds sighted. To account for the fact that some locations are visited by bird watchers more often than others, we averaged the number of birds sighted per location per observer. Outliers with extreme values were removed and kriging, a method of spatially weighted averaging, was used to estimate the mean number of birds sighted at each county centroid.

Land cover data was obtained from the National Land Cover Database (NLCD), a national dataset that characterizes landcover status and changes from 2001, 2006, 2011, and 2019. Linear interpolation was performed to assess land cover information for the years for which NLCD was not available before 2019, and linear extrapolation was used for the missing data for the years after 2019. For each county, the proportion of that county’s land mass which fell into each of 14 NLCD categories was computed. For information on how these NLCD categories are defined, please see the US Geological Survey’s related NLCD website: https://www.usgs.gov/centers/eros/science/national-land-cover-database.

Our analysis accounted for el Niño, an unusual warmth pattern in international climate, presence, and absence across the timeline. El Niño years were collected from the National Oceanic and Atmospheric Administration’s Climate Prediction Center. We created a binary “el Niño” variable as a 0 or 1 value for el Niño year and included the variable in our final dataset.

County level data on habitat suitability for *Culex quinquifasciatus*, *Culex pipiens*, and *Culex tarsalis* were obtained from data provided by Gorris et al (2021), and was assumed to be static across all study years [37].

Previous work has shown that WNV cases in Texas tend to exhibit a three-year cycle of increase and decrease [38]. As the presence of such a pattern in one state suggests that it might be present elsewhere, indicator variables for cycle-year (cycle year 1, cycle year 2, or cycle year 3 (reference level)) were included in the model to assess the predictive utility of such cycle [38]. Although there is evidence of seasonality in WNV transmission, our data set included only annual counts, and seasonality was not necessary to include in our model [39, 40].

### The model

We used a Bayesian negative binomial regression model, which can be described via the following equations:

$$Y_{st}|\mu_{st},\sigma^{2}∼NB(\mu_{st},\sigma^{2})$$ (1)

$$\mu_{st}=exp(\eta_{st})$$ (2)

$$\eta_{st}=\beta_{0}+\beta x_{st}+\phi_{t}+\psi_{s}$$ (3)

$$\phi =(\phi_{1},\ldots ,\phi_{T})∼AR(1)$$ (4)

$$\psi =(\psi_{1},\ldots ,\psi_{S})∼CAR$$ (5)

The number of cases in county s and year t are signified by *Y*_*st*_, and *Y*~*NB*(*μ*,*σ*^2^) indicates that the random variable *Y* follows a negative binomial distribution with mean *μ* and dispersion parameter *σ*^2^. The mean *μ*_*st*_ is related to a linear *η*_*st*_ which is a function of covariates; *σ*^2^ is a non-negative dispersion parameter. Four pieces compose *η*_*st*_: the shared intercept term *β*_0_, covariate factors ***x***_*st*_, a temporal effect *ϕ*_*t*_, which allows mean counts to change over time, and a spatially varying intercept *ψ*_*s*._

In the Bayesian paradigm, all parameters are random variables and have prior distributions. The spatially varying intercepts (***ψ***) is assumed to follow a conditional auto-regressive (CAR) prior to ensure that intercepts from counties that are close in space have similar values, as counties that are adjacent to each other often have similar incidence rate. More formally, *ψ*~*N*(**0**,*Q*^−1^) where **0** is the *S*-dimensional zero vector and *Q* is an *S*×*S* matrix with diagonal entries given by *Q*_*ss*_ = *τ*(*n*_*s*_+*d*) and off-diagonal entries given by *Q*_*sr*_ = −*τ* if counties *s* and *r* share a border and *Q*_*sr*_ = 0 otherwise. Here *n*_*s*_ is the number of neighbors of county *s*, log*τ* follows a loggamma(0,0.0005) prior distribution and log(*d*) follows a loggamma(1,1) prior distribution. For more on CAR models, see Banerjee et al. 2003 or Besag 1974 [41, 42]. The temporal effects (***ϕ***) are assumed to follow a mean 0 autoregressive prior distribution of order 1 with precision *γ* and lag 1 autocorrelation *ρ*, where log(*γ*) follows a loggamma (1,0.00005) prior distribution and $log(\frac{\rho }{1-\rho })$ follows a normal(0,0.15) prior. The intercept (*β*_0_) and regression coefficients (*β*) were assigned independent normal priors with mean 0 and precision 0.001. The dispersion parameter (*σ*^2^) follows a penalized complexity gamma (1/a, 1/a) prior specification with base model a = 0 implemented by the R INLA package [33].

### Model selection

Once all data on covariates were collected, tests for multicollinearity were performed. Variables with the highest variance inflation factor (VIF) were eliminated until every VIF was below 5 [43]. This resulted in removing the variable proportion of land mass in cultivated crop land cover. We then fit the model using all the remaining variables. We then tested for spatial autocorrelation using a likelihood ratio test from a CAR model fit using the spautolm function of the R package spatialreg [44]. This test indicated significant spatial autocorrelation and motivated our use of a CAR prior for the spatially varying intercepts. Next, backwards selection was performed. In each step, we removed the variable whose elimination resulted in the largest decrease in DIC until there were no more variables whose removal decreased DIC. Variables chosen for the final model included: proportion of those above 65, elevation in feet, proportion of land mass in open water, developed medium intensity (suburban environments), developed high intensity (urban environments), deciduous forest, evergreen forest, mixed forest, herbaceous vegetation, woody wetlands, and emergent herbaceous wetlands, habitat suitability for *Culex quinquefasciatus*, *Culex pipiens*, and *Culex tarsalis* mosquitos, house sparrow, blue jay, and American crow host populations, a binary variable for el Niño year, year, and cycle variables. All continuous variables were standardized. We considered having a population offset term; however, including the term resulted in greater error in prediction performance. Because of this, we opted to not include an offset term in our final model. An integrated nested Laplace approximation (INLA) model in R software was used to fit our model [33]. This provided a more efficient way to fit our model than standard Markov chain Monte Carlo methods. Absolute error was calculated in our models by the following formula:

$$e_{st}=|exp(\eta_{st})-y_{st}|$$

where *e*_*st*_ signifies the absolute value of the prediction error for county *s* and year *t*.

Additionally, all map figures were created through ArcGIS Pro 2.8.7. The predictive power of our final model was assessed by predicting counts for each year between 2010 and 2021 using data up to but not including that year and comparing the predicted counts to the observed counts for each year.

## Results

Model selection was performed using holdout data for the year 2021. After model selection, our final model was able to predict 2021 cases with a median absolute prediction error of 0.0629 cases. The posterior mean estimates, standard deviations, and 95% highest posterior density interval bounds for the regression coefficients for our final model can be found in Table 1. Plots of the estimated posterior probability distribution for select parameters can be found in Web Appendix A (S1 File) of our supplementary material.

**Table 1:** Estimation of regression coefficients.

Variable	Estimate	Standard Deviation	Lower Bound of 95% Highest Posterior Density Interval	Upper Bound of 95% Highest Posterior Density Interval
Intercept	-3.16E+00	6.07E-01	-4.36E+00	-1.96E+00
Proportion Age Over 65*	-3.33E-01	3.05E-02	-3.92E-01	-2.73E-01
Elevation (feet)	-5.89E-02	1.04E-01	-2.62E-01	1.44E-01
Open Water	-7.94E-02	4.59E-02	-1.69E-01	1.04E-02
Developed Medium Intensity (Suburban)*	4.60E-01	4.75E-02	3.67E-01	5.53E-01
Developed High Intensity (Urban)*	-1.44E-01	4.23E-02	-2.27E-01	-6.08E-02
Deciduous Forest	-1.10E-01	7.95E-02	-2.66E-01	4.54E-02
Evergreen Forest*	-1.33E-01	5.55E-02	-2.42E-01	-2.44E-02
Mixed Forest	2.03E-02	5.49E-02	-8.74E-02	1.28E-01
Herbaceous Vegetation	6.15E-02	5.24E-02	-4.13E-02	1.64E-01
Woody Wetlands	-3.19E-02	5.66E-02	-1.43E-01	7.90E-02
Emergent Herbaceous Wetlands	4.53E-03	3.66E-02	-6.72E-02	7.60E-02
Sparrow	-4.07E-03	1.22E-02	-2.80E-02	1.98E-02
Jay	8.21E-03	7.73E-03	-6.54E-03	2.37E-02
Crow	1.60E-02	1.07E-02	-5.02E-03	3.70E-02
El Nino	2.95E-01	5.06E-01	-6.98E-01	1.29E+00
Culex Quinquefasciatus Suitability*	6.78E-01	8.60E-02	5.09E-01	8.46E-01
Culex Pipiens Suitability*	3.57E-01	7.29E-02	2.14E-01	4.99E-01
Culex tarsalis Suitability	8.52E-02	1.08E-01	-1.26E-01	2.96E-01
Cycle 1 Variable	-3.31E-01	5.39E-01	-1.39E+00	7.26E-01
Cycle 2 Variable	-2.32E-01	5.41E-01	-1.29E+00	8.27E-01
Year	5.84E-01	4.73E-01	-3.42E-01	1.54E+00

*Found Statistically Important

We then compared the fitted values with the observed counts for each year. A handful of counties had a mean absolute prediction error greater than 15 counts. This represented a skewed error distribution and therefore we reported median absolute prediction error. For each year from 2010 to 2021, we withheld the data from that year, fit the model and computed the absolute prediction errors (*e*_*st*_*s*) for that year. We report the median absolute prediction error for years 2010–2021, and counties with over 100 count difference between observed and expected per year, in Table 2. The prediction model performed well for most of the counties, with only 83 (2.67%) counties with an absolute error of two counts or higher for the year 2021. The counties with the largest errors tended to be the most highly populous counties.

**Table 2:** Absolute value of prediction error and counties with highest error, by year.

Year	Absolute Prediction Error (Cases)	Absolute Prediction Error (Incidence per 100,000)	Counties with Error > 100 Counts
2010	4.99E-02	5.05E-02	-
2011	8.30E-02	8.34E-02	-
2012	3.17E-02	3.16E-02	Dallas, TX; Cook, IL; Tarrant, TX
2013	8.02E-02	7.94E-02	-
2014	2.17E-01	2.13E-01	Cook, IL; Orange, CA
2015	9.26E-02	9.03E-02	Los Angeles, CA
2016	7.18E-02	6.96E-02	-
2017	9.18E-02	8.84E-02	Los Angeles, CA
2018	1.61E-01	1.54E-01	Los Angeles, CA
2019	9.35E-02	8.91E-02	-
2020	5.84E-02	5.55E-02	-
2021	6.29E-02	5.88E-02	Maricopa, AZ

**Fig 1:**
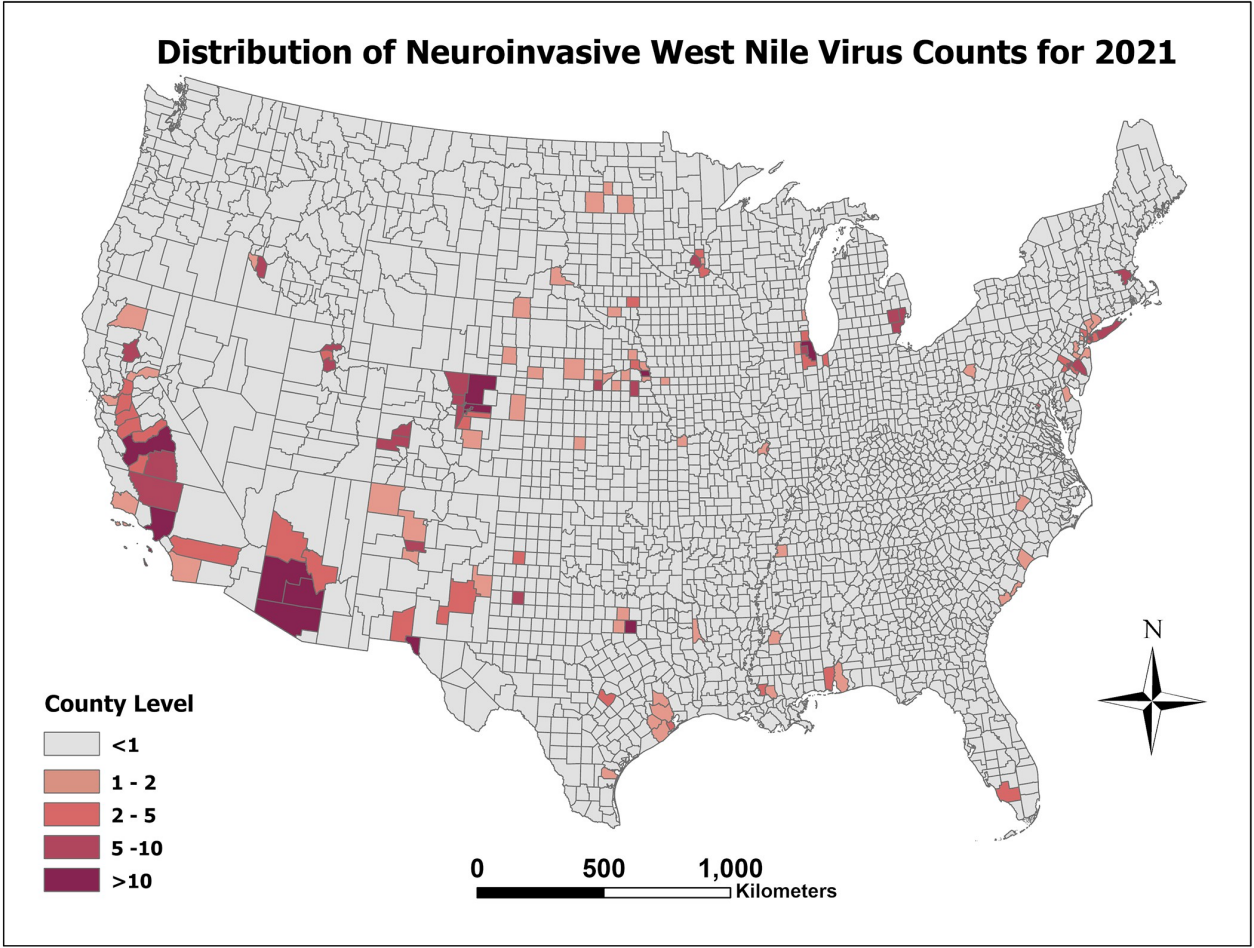
Distribution of observed WNND incidence per 100,000 for 2021.

**Fig 2:**
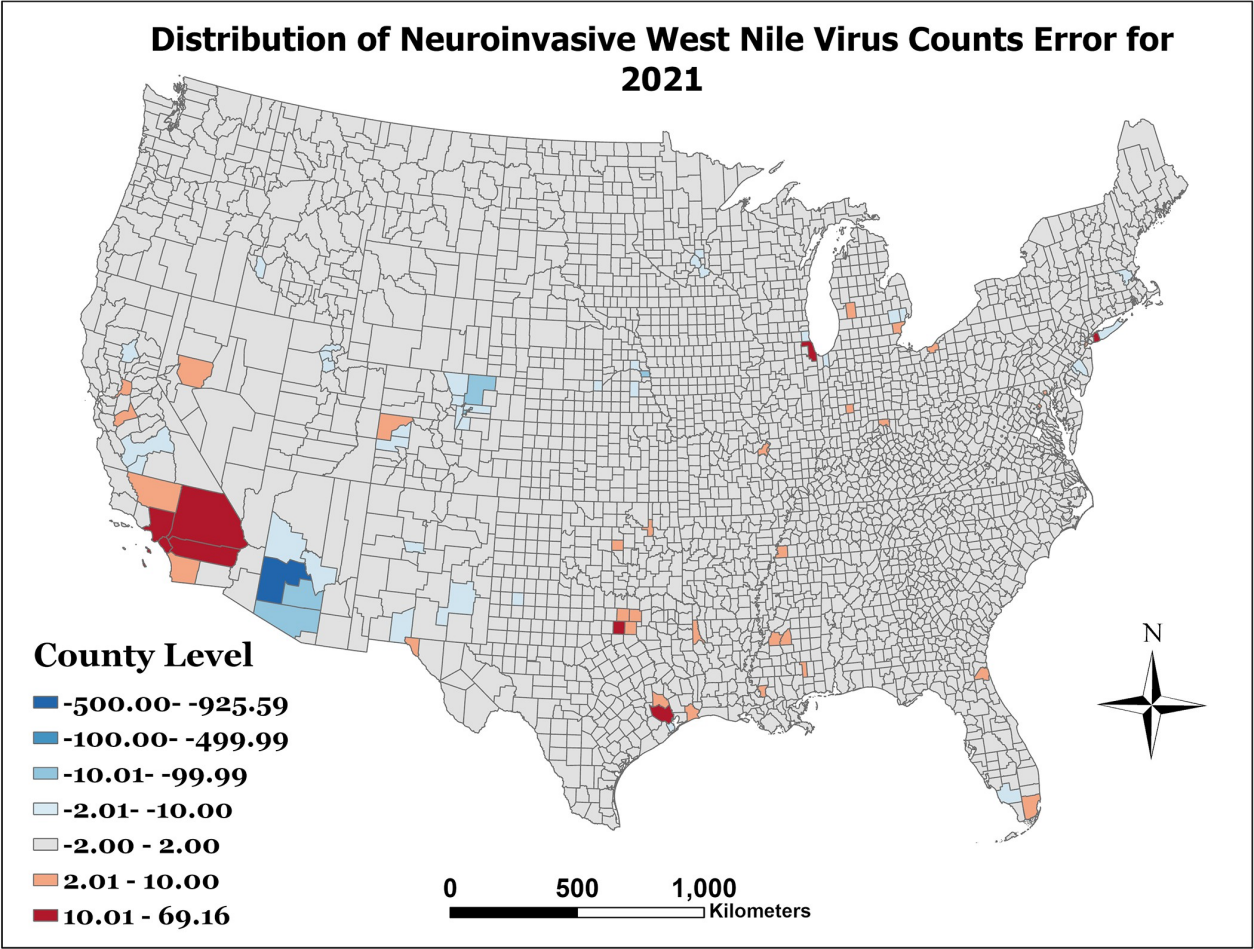
Distribution of the observed WNND counts and the predicted WNND counts for 2021.

**Fig 3:**
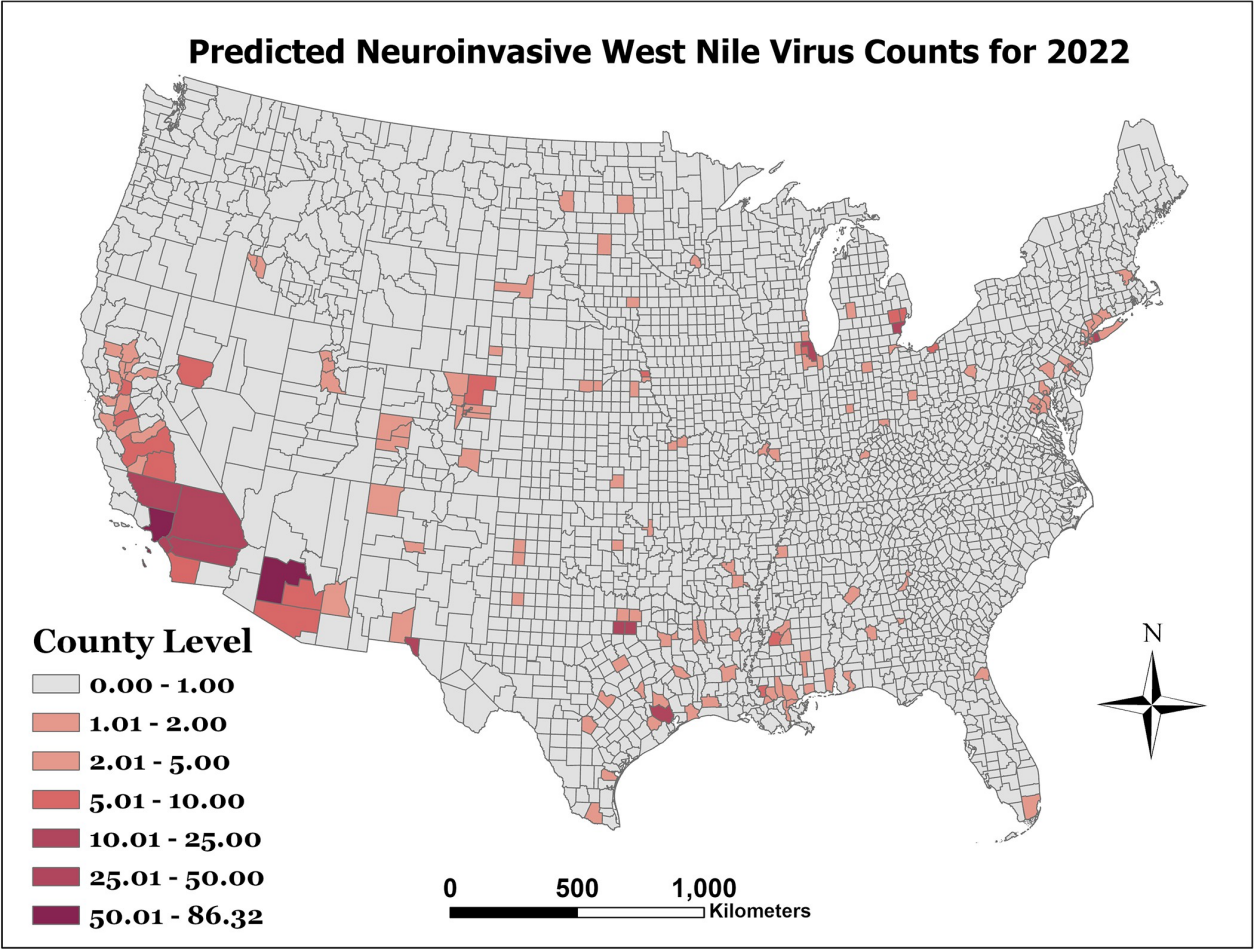
Predicted WNND counts for 2022.

**Fig 4:**
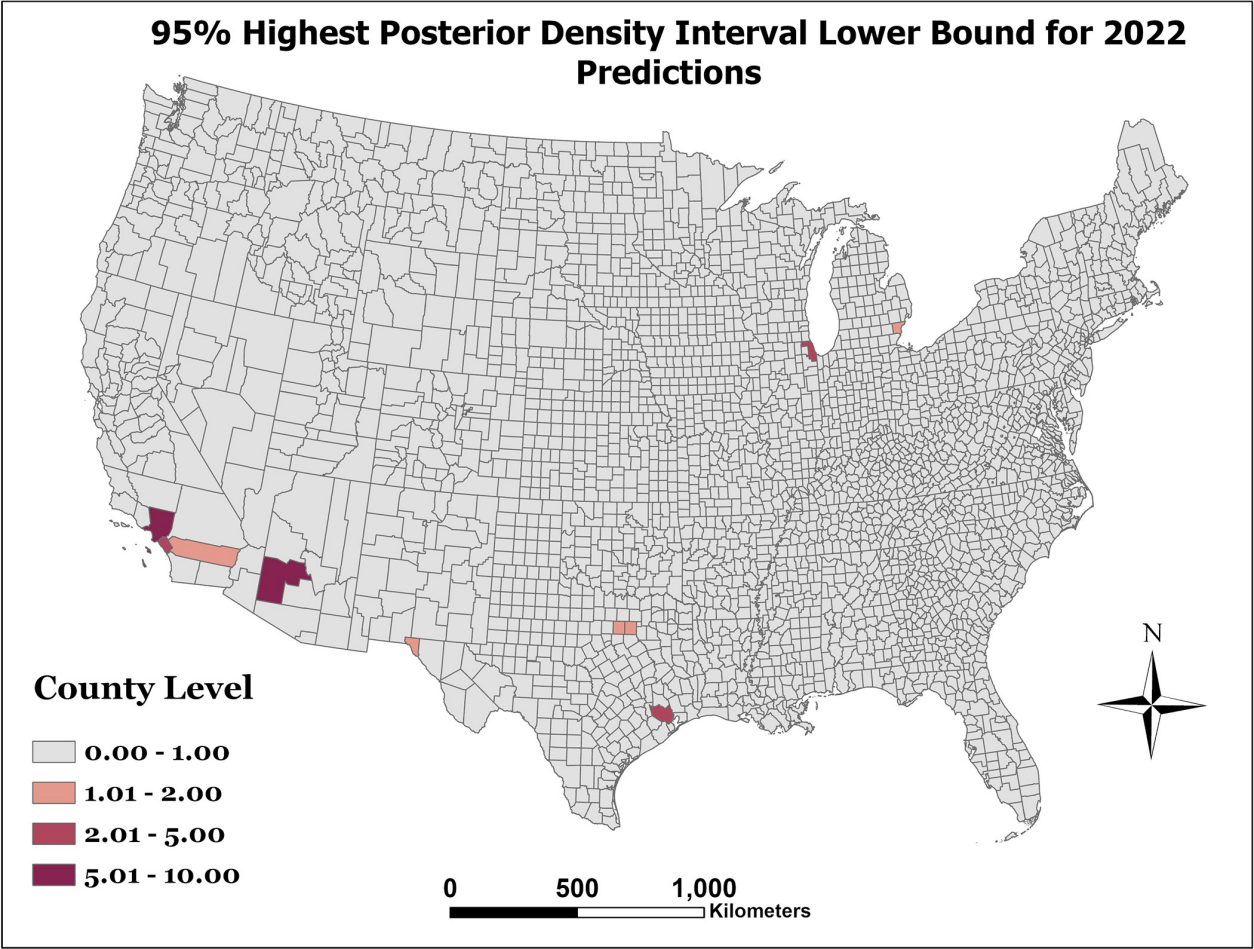
95% highest posterior density interval lower bound for 2022 predictions.

**Fig 5:**
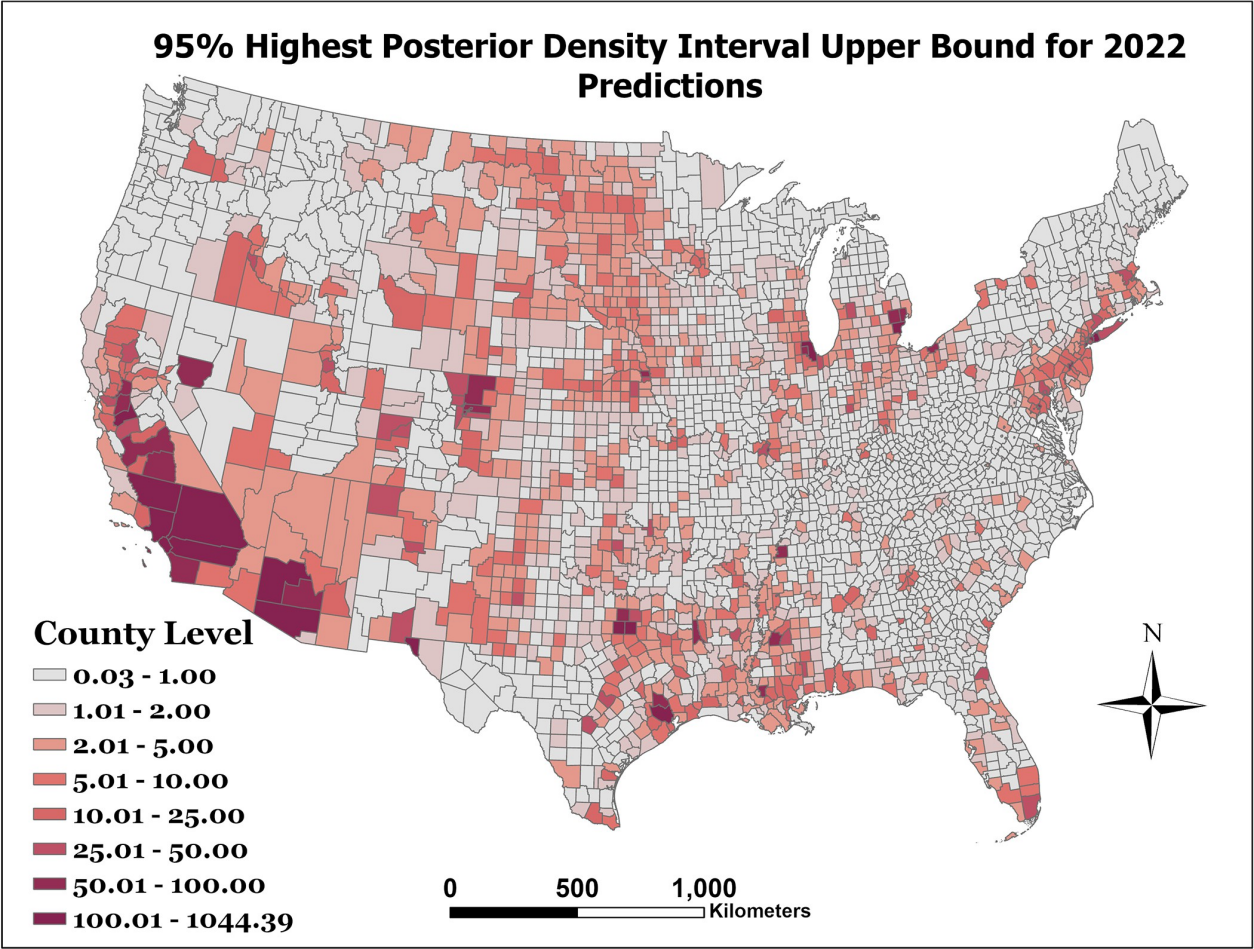
95% highest posterior density interval upper bound for 2022 predictions.

Fig 1 presents the observed WNND incidence per 100,000 for the year 2021. As can be seen in Fig 2, which represents the prediction error as the predicted counts minus the observed counts (e.g exp (*η*_*st*_)−*y*_*st*_), there is limited error among most counties. However, our model can be improved in counties with large populations. Finally, after model prediction power was assessed with the holdout data, the model was then fit to the entire dataset to make predictions for 2022. The distribution of this prediction can be seen in Fig 3. The lower and upper bounds of the 95% prediction highest posterior density credible interval for the 2022 predicted counts can be seen in Figs 4 and 5, respectively.

As shown in Figs 3 through 5, the counties with the highest predicted WNND incidence were Los Angeles County, CA; Harris County, TX; Orange County, CA; Maricopa County, AZ; and Cook County, IL, all of which have a population greater than 2 million, and among which are the four are the most populous counties in the United States of America.

## Discussion

The inclusion of predictive models into public health planning can guide government organizations to target high risk areas for prevention efforts and resource allocation for education programs, mosquito surveillance, and human arboviral disease surveillance. Funding for public health programs, particularly for vector-borne diseases, is often reactive to outbreaks with funding arriving after novel disease introduction or towards the outbreak decline [18]. Our WNND prediction model, and others like it, provide support for the utility of forecast arboviral modelling as a feasible means to aid decision makers on vector-borne diseases that can be endemic, yet subject to large outbreaks [22, 27, 45, 46]. The WNND model presented here is distinct in its ability to employ a Bayesian model, human WNND case counts, and novel ecological/life cycle variable additions to generate a county-level national forecasts for real-time prediction.

While our model was not designed to assess causal relationships, and we did not look at the effect of potential mediation within the variables in the model, we did find associations between certain input variables and WNND counts that are biologically plausible. Please note, all continuous variables were standardized, which allowed us to directly assess the magnitude of coefficients. Developed medium intensity landcover, often associated with suburban areas, was found to have a positive association with WNND counts. This could be due to an environment where mosquitos can easily breed in lawns and standing water, while having a multitude of humans to infect. In contrast, developed high intensity, which can be surmised as concentrated urban areas, was found to have a negative association with WNND counts. Although there are large populations in cities, there is limited still water or green space for mosquitos to inhabit and breed, making the transmission of WNV less likely. Further, studies suggest the existence of potential mosquito species competition between *Aedes* spp and *Culex* spp in urban environments, with *Aedes* spp being more likely to outcompete *Culex* spp for resources and survival [47, 48]. Lastly, *Culex quinquefasciatus* and *Culex pipiens* habitat suitability were found to be positively associated with higher WNND counts. This coincides with literature supporting that these two *Culex* vectors serve as the principal species for disease transmission [5, 6].

In contrast, we found two input variables had alternative influences contradictory to the scientific literature. Proportion 65 years of age and above was found to have a negative relationship with WNND counts. This is contrary of what is to be expected, as geriatric age is a known risk factor for WNND [49]. Similarly, developed high intensity landcover and evergreen forest cover were found to have a negative association with WNND counts—an unexpected finding as lower temperatures created by tree canopy are typically associated with greater *Culex* mosquito populations [50–52]. The authors hypothesize the negative association of forest cover could be an effect of rural communities having less access to healthcare and thus, fewer diagnoses and reports of WNND.

Our 2022 prediction results estimated WNND counts clustered around the southwest USA, and interspersed throughout the remaining counties. This coincides with most yearly WNND patterns for the last decade, though 2020 showed a sparse dispersion of WNND counts. Though the 2022 highest counts cover a concentrated area within the southwest, the model predicted small counts for most of the remaining counties.

Multiple techniques have been successfully used in arboviral disease forecast modelling, including machine learning [53], neural networks [54], and Bayesian models [27, 32]. One advantage of the Bayesian approach over others is that it provides probability distributions for all unknown parameters, which facilitates seamless inference on predicted counts. This manuscript, using Bayesian inference, is one of the first studies to predict mosquito borne disease in humans for the entire contiguous USA at the county level. Because our model was able to predict cases in 97.33% of counties with a margin of error of less than 2 counts, future studies could explore using a similar model to predict non-neuroinvasive WNV incidence in addition to neuroinvasive, or other arboviruses such as dengue virus. Avian vector data has great potential to predict other diseases such as Crimean-Congo hemorrhagic fever virus and tick-borne encephalitis virus. Our model included a yearly-fluctuating el Niño variable, but future studies could benefit by developing a more dynamic model over the course of the year including weather data and additional seasonal patterns [55].

Though our model performed well in predicting WNND cases in the contiguous USA, it is not without limitations. Diagnoses and reporting of arboviruses are often incomplete or underreported, therefore counts of WNND could be underestimated for this study. Avian host data was citizen collected and therefore was not consistent across locations in the USA, and may not be representative of all counties. Furthermore, the locations at which birding data was collected were not randomly selected, but are likely skewed towards locations with more birds, as bird watchers tend to favor these locations. Therefore, this data may not perfectly capture the distribution of the bird populations. However, we standardized this data for the number of birders in a particular location by averaging the number of bird sighting at a location over the number of birders present.

Additionally, the CDC case data was aggregated to the yearly scale, as opposed to weekly or monthly. WNND exhibits a distinct seasonal pattern with most cases occurring in warmer months [56, 57]; as our model makes predictions on a yearly scale, it did not account for this seasonality. Furthermore, without more temporally granular case data, we could not evaluate the impact of short-term weather fluctuations on WNND cases counts. Future studies would benefit from assessing this.

The use of backwards elimination to perform model selection is not without limitations, and different model selection procedures will often produce different final models [58, 59]. To give a sense of the uncertainty inherent in model selection, we provide the results from an alternative model resulting from a different model selection procedure in Web Appendix B (S1 File).

Though accurate in most counties, our model tended to have larger error in counties with high populations. Adding an offset term did not substantially improve the model. This suggests the relationship between WNND counts and population is complex and non-linear. However, including non-linear population variables in the model also did not improve performance in highly populous counties. This underprediction may be related to the substantial underreporting of WNND cases. Highly populated, urban counties tend to have better access to healthcare and hence we would expect a lower rate of unreported WNND cases per person in urban counties than in rural ones. Enhanced reported of cases in highly populous counties may explain why those counties reported more cases than predicted by the model.

It should be noted that this model was used to predict cases of neuroinvasive WNV rather than WNV febrile or asymptomatic cases. As cases of WNND are typically severe, WNND is significantly more likely to be diagnosed than febrile cases of WNV [57]. The substantial underdiagnosis of febrile WNV would result in a great amount of uncertainty in our model, thus producing less accurate predictions. Additionally, assessing WNND counts does have benefits. Targeting areas of high WNND risk over high WNV risk allow stakeholders to focus limited intervention resources to prevent the most severe forms of the disease. Additionally, because WNND data are more accurate, researchers can identify high risk WNND areas more easily.

In closing, this study sought to predict cases of neuroinvasive WNV at the county level for the contiguous USA states using a spatio-temporal Bayesian negative binomial regression. An integrated nested Laplace approximation approach was used to fit the model, saving extensive computational cost. After variable selection, the model performed well and in predicting historical WNND cases in 97.33% of contiguous USA counties, though the model can be improved on counties with very large populations. These findings have implications for future stakeholder decisions and interventions regarding targeting areas for WNV prevention.

## Supporting information

S1 File Additional analysis outputs, additional maps, and alternative model results. This supplemental file is comprised of four distinct appendices that provide detailed model parameters, data availability sources, additional figures, and results from an alternative model selection procedure.
(PDF)
