# Supplementary material for: The utility of a Bayesian predictive model to forecast neuroinvasive West Nile virus disease in the United States of America, 2022
Source: PLoS One. 2023 Sep 8;18(9):e0290873. doi: 10.1371/journal.pone.0290873 (PMC10490885; doi:10.1371/journal.pone.0290873)
Supplement: S1 File — This supplemental file is comprised of four distinct appendices that provide detailed model parameters, data availability sources, additional figures, and results from an alternative model selection procedure. (PDF) [file pone.0290873.s001.pdf]

**Supplementary File 1. Additional analysis outputs, additional maps, and alternative model results.**

Web Appendix A – Posterior Probability Distributions of Select Model Parameters

Plots of the estimated posterior probability distribution for the intercept ($\beta$) and regression coefficients ($\boldsymbol{\beta}$) can be found in Web Figure 1:

Web Figure 1: Estimated posterior probability distribution functions for the intercept ($\beta$) and regression coefficients ($\boldsymbol{\beta}$)

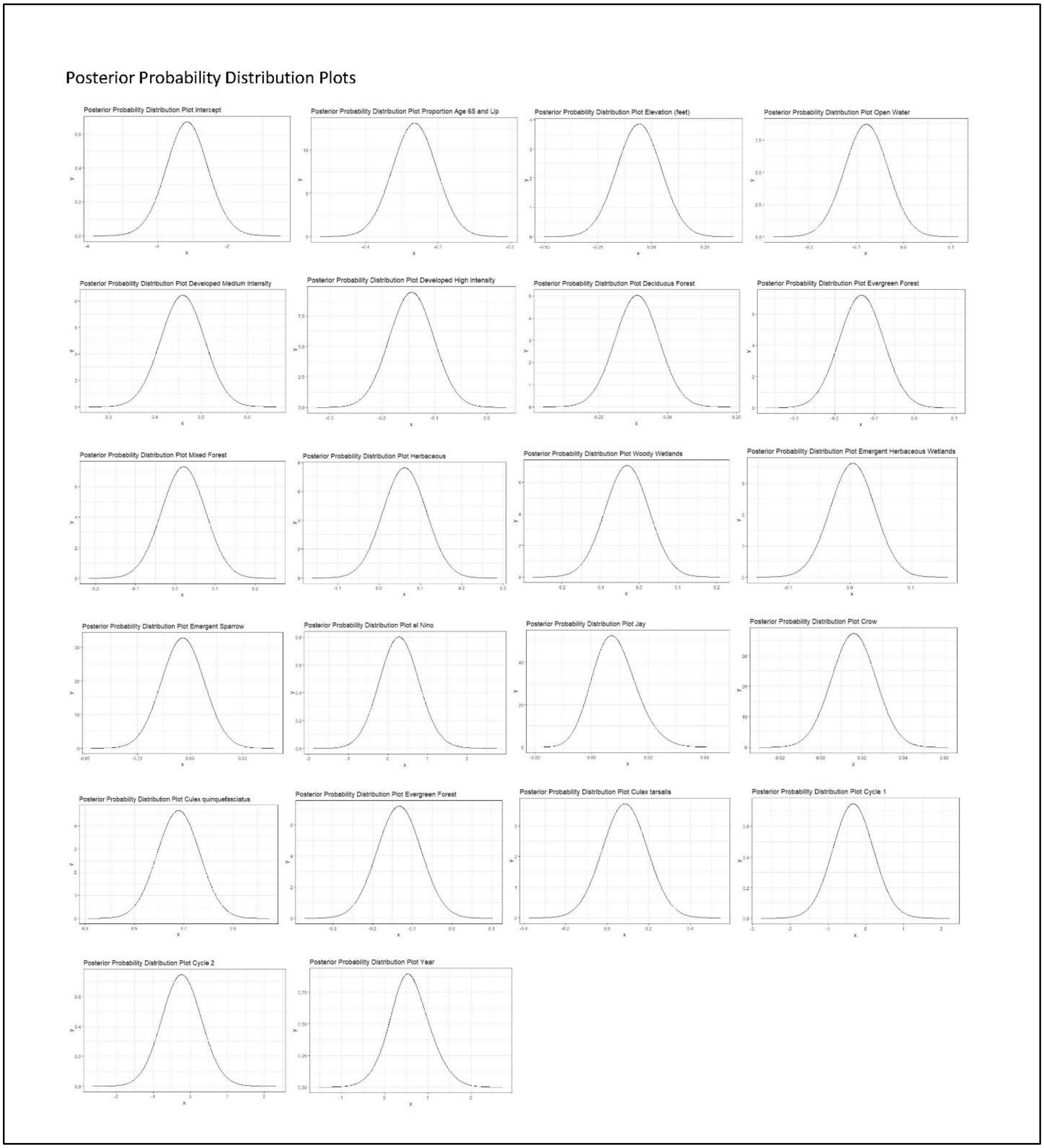

A plot of the estimated posterior probability distribution for the dispersion parameter ($\sigma^2$) is provided in Web Figure 2.

Web Figure 2: Estimated posterior probability distribution function for the dispersion parameter ($\sigma^2$).

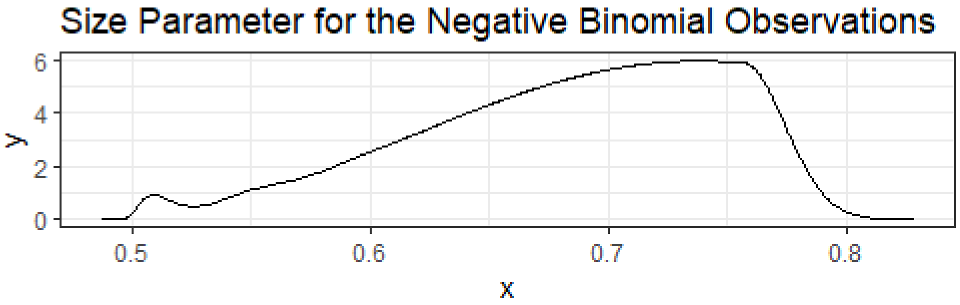

A plot of the estimated posterior probability distributions for the temporal random effects ($\boldsymbol{\phi}$) are provided in Web Figure 3.

Web Figure 3: Estimated posterior probability distribution functions for the temporal random effects ($\boldsymbol{\phi}$).

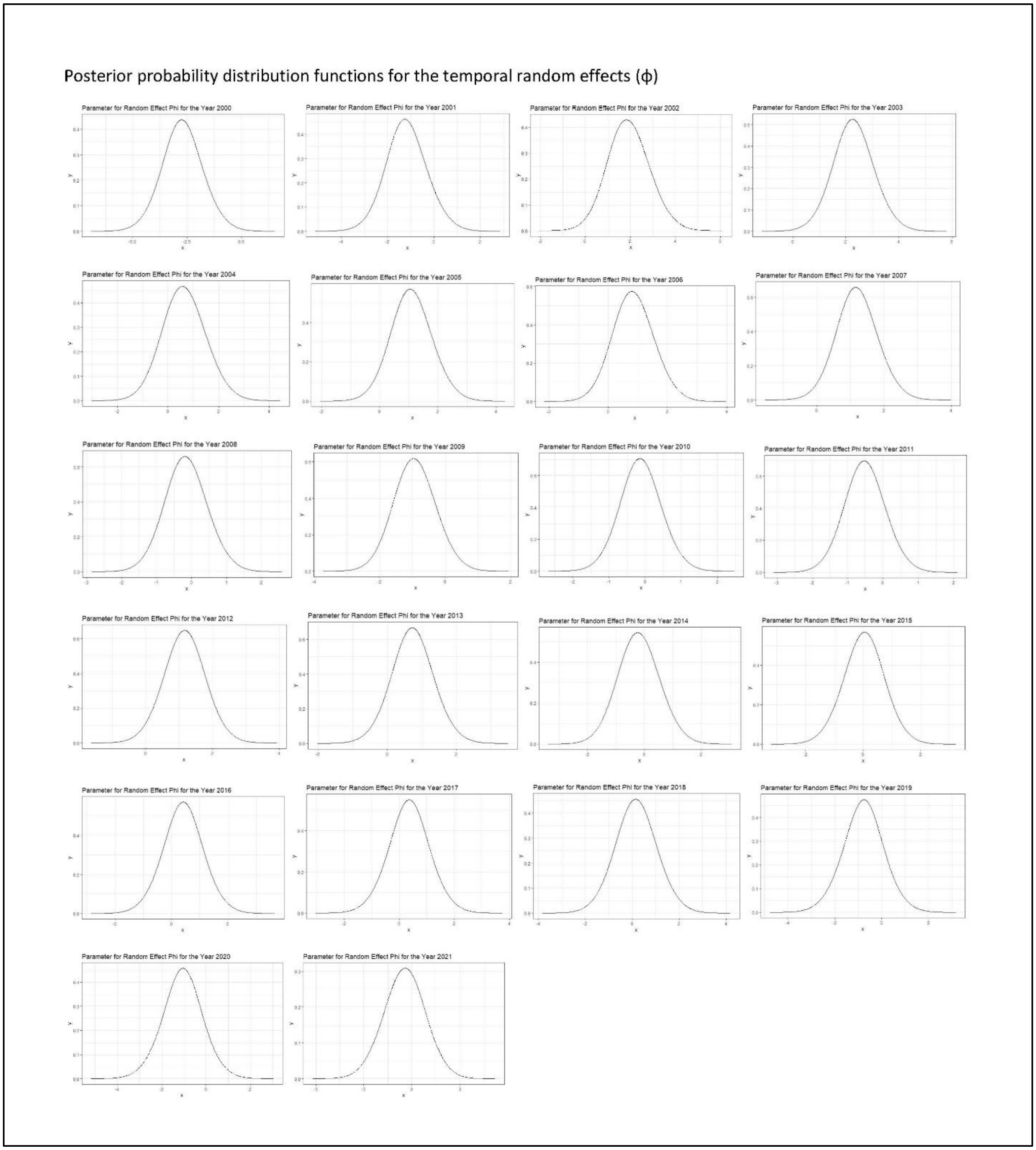

Plots of the estimated posterior probability distribution for the spatial random effects ($\boldsymbol{\psi}$) for 10 randomly selected counties are provided in Web Figure 4:

Web Figure 4: Estimated posterior probability distribution functions for the spatial random effects ($\boldsymbol{\psi}$) for 10 randomly selected counties.

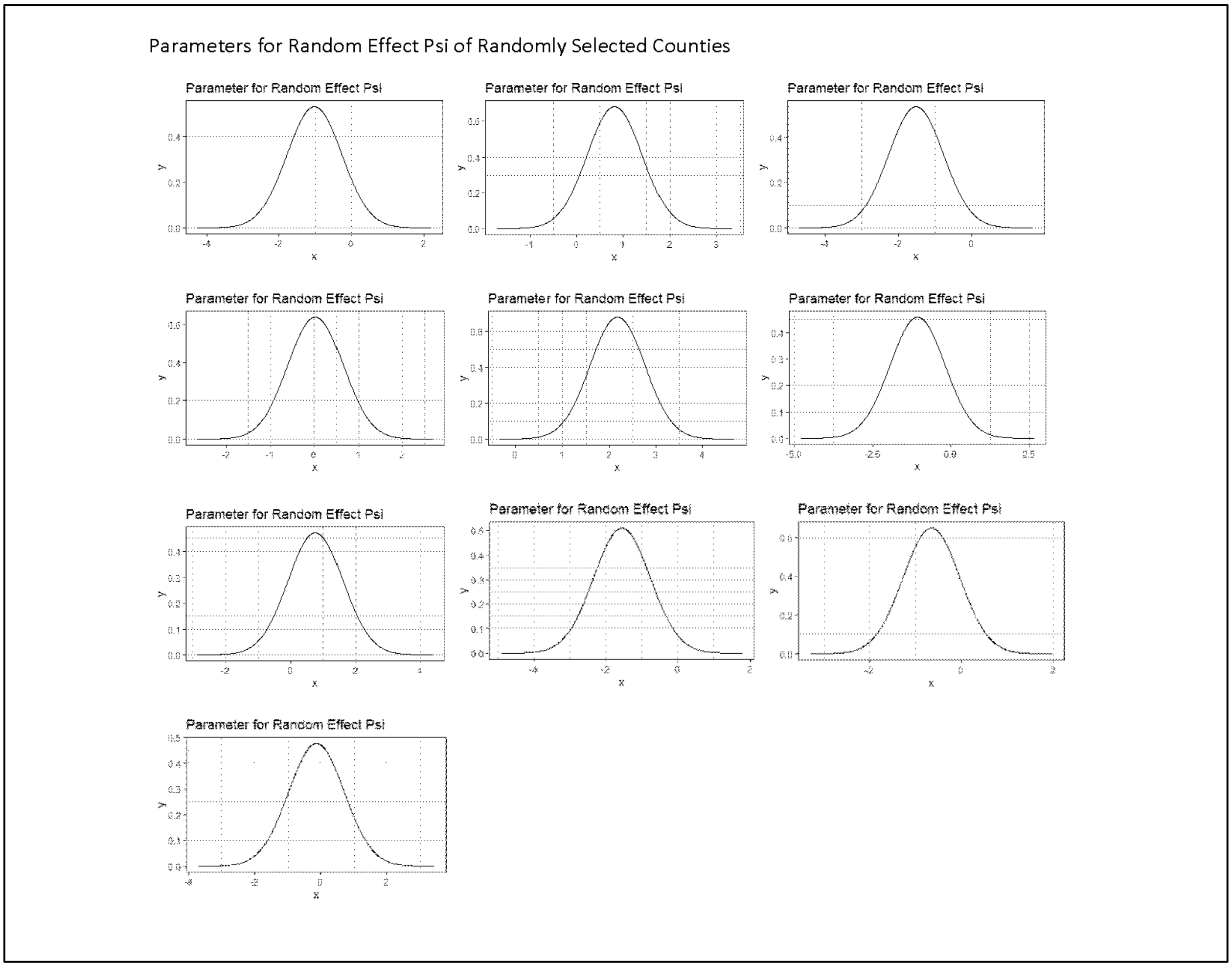

Web Appendix B: Predicted Neuroinvasive West Nile Virus Counts for 2022 from the Alternate Model

The alternate model was selected by running the full model and assessing the 95% credible intervals. Variables whose 95% credible interval included 0 were considered statistically non-important and were deemed potentially removable from the model. The standardized absolute value was then computed for each statistically non-important variable; this was calculated as the absolute value of the posterior mean divided by the posterior standard deviation. The variables with the smallest standard absolute values were removed one at a time, and the model rerun without the variable. This was repeated until only statistically important variables remained in the model. The 2022 predicted WNND counts from this alternate model are shown in Web Figure 5. Web Figure 5: The 2022 Predicted WNND Counts from the Alternate Model

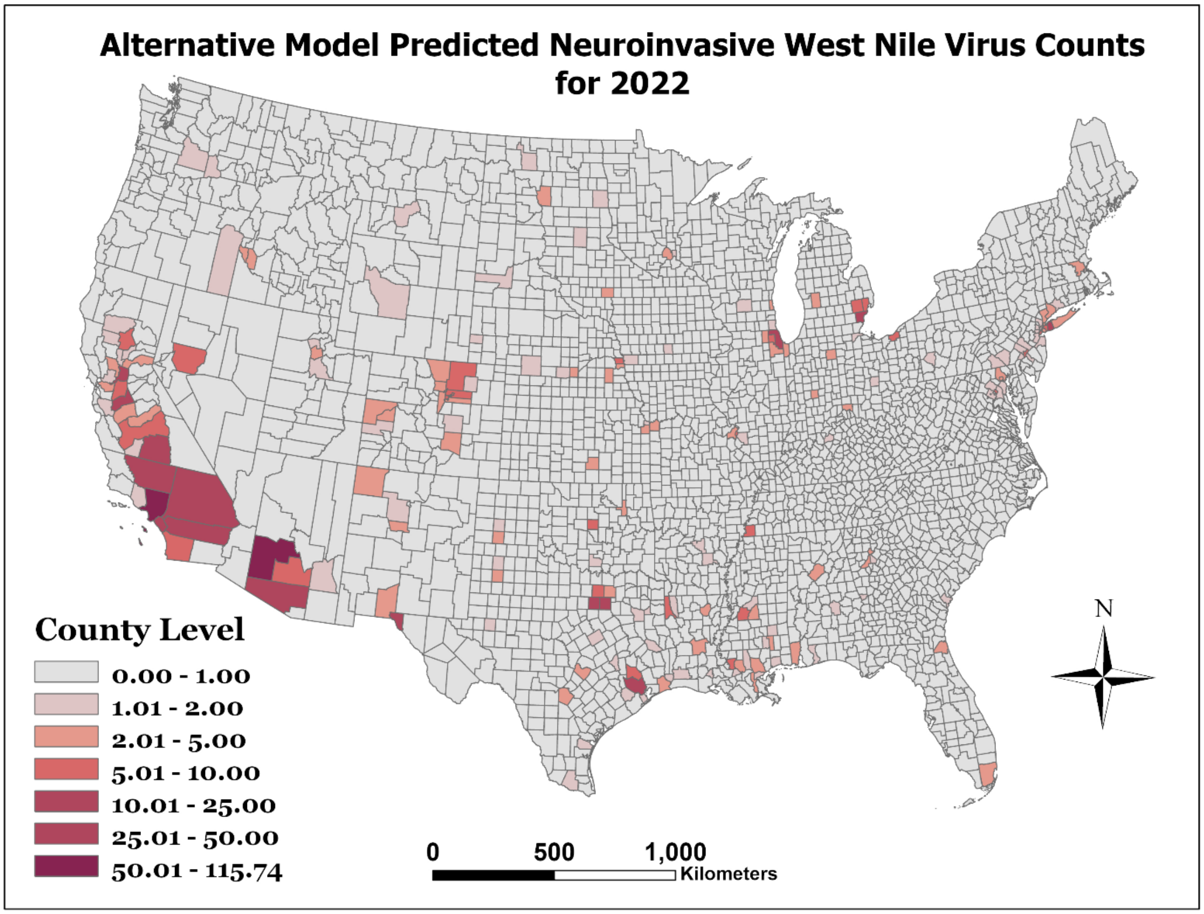

Web Appendix C: Additional Figures

The estimated 2021 population of each county is shown in Web Figure 6.

Web Figure 6: Total Population Estimate of the Contiguous United States Counties for 2021

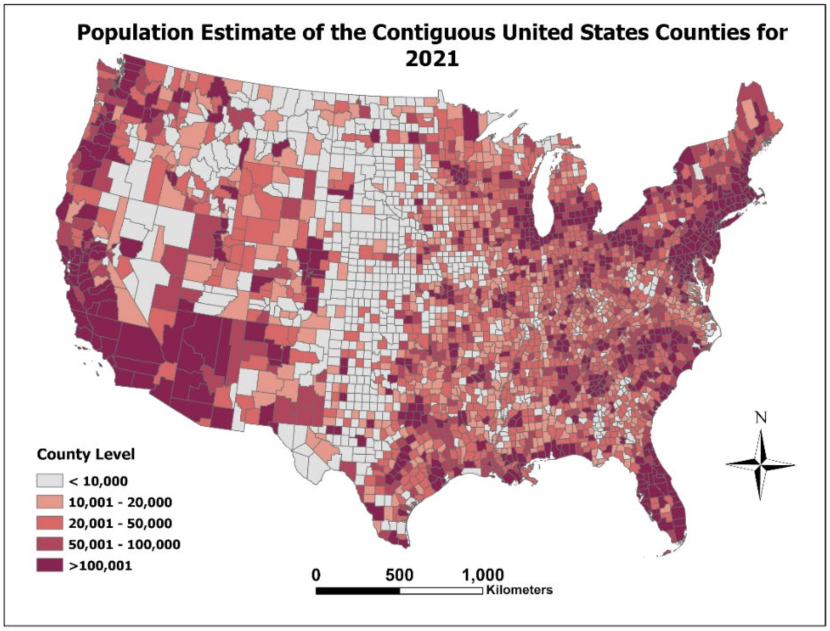

Web Figure 7 depicts the **predicted 2022 WNND counts** from the final model minus the **observed WNND counts from 2021** to facilitate comparisons of the 2022 predictions to the patterns observed the previous year.

Web Figure 7: The map depicts the predicted 2022 WNND counts from the final model minus the observed 2021 WNND counts.

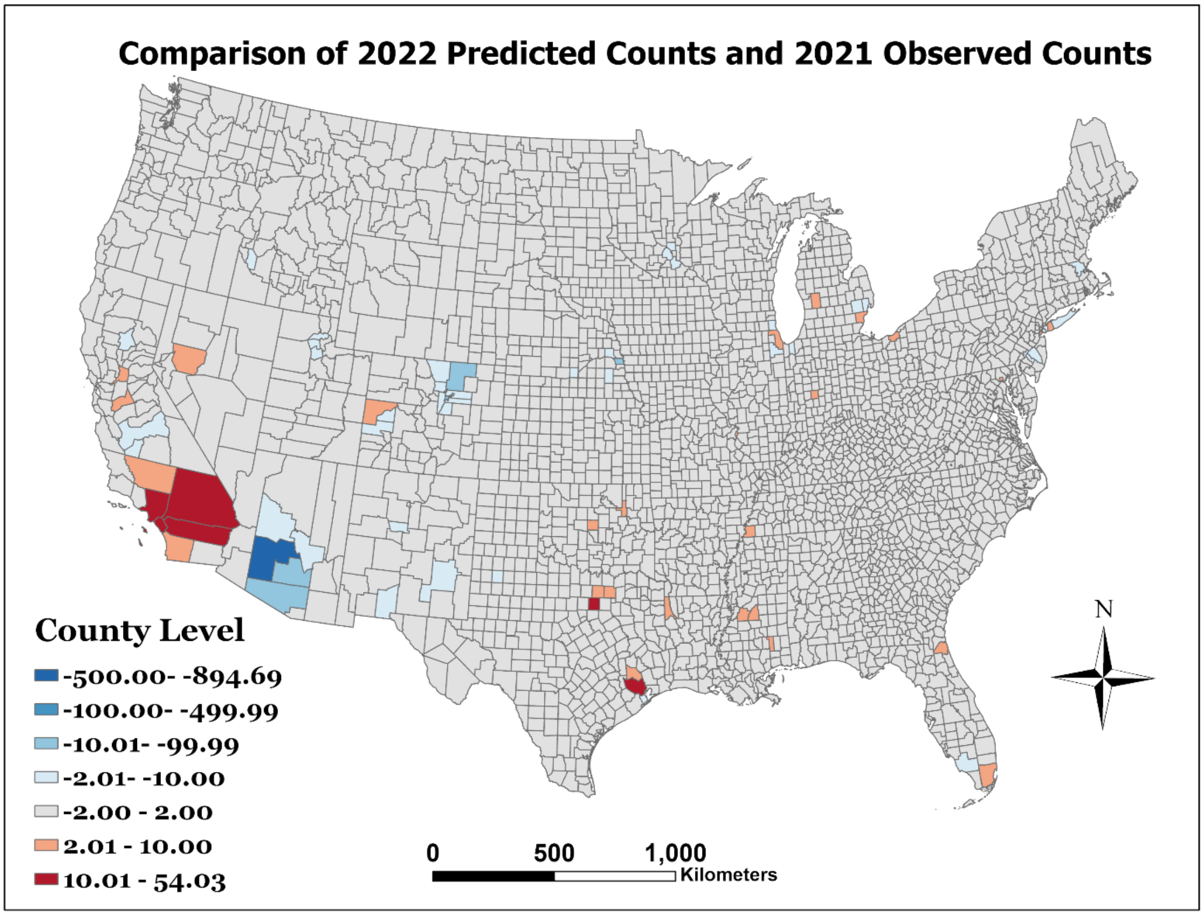

Observed incidence per 100,000 for 2021 was calculated by dividing the number of WNND cases reported in each county in 2021 by the population of the county and multiplying by 100,000.

Web Figure 8: Distribution of Observed Neuroinvasive West Nile Virus **Incidence per 100,000** for 2021

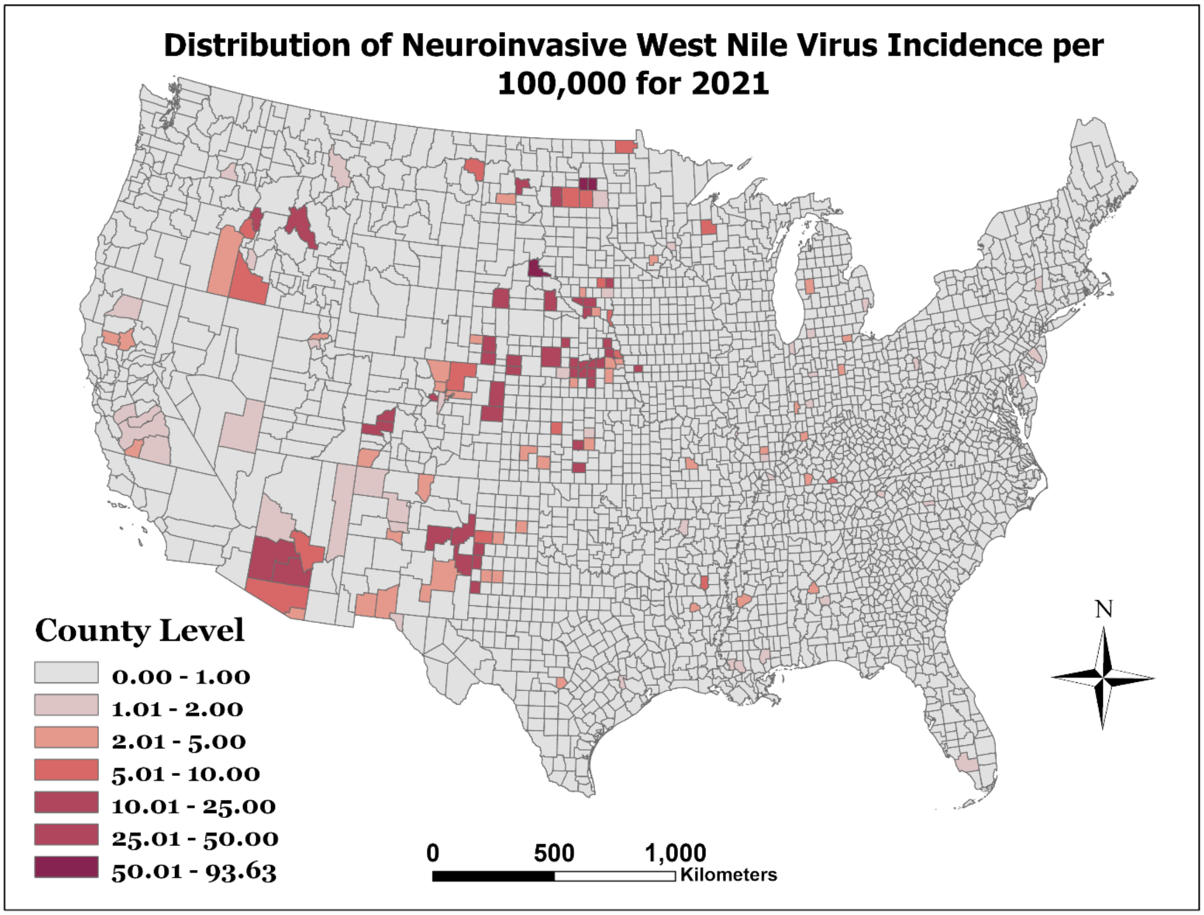

Predicted neuroinvasive West Nile virus incidence per 100,000 for 2022 was calculated by dividing the predicted WNND count for each county for 2022 from the final model by the population of the county and multiplying by 100,000.

Web Figure 9: Predicted Neuroinvasive West Nile Virus **Incidence per 100,000** for 2022

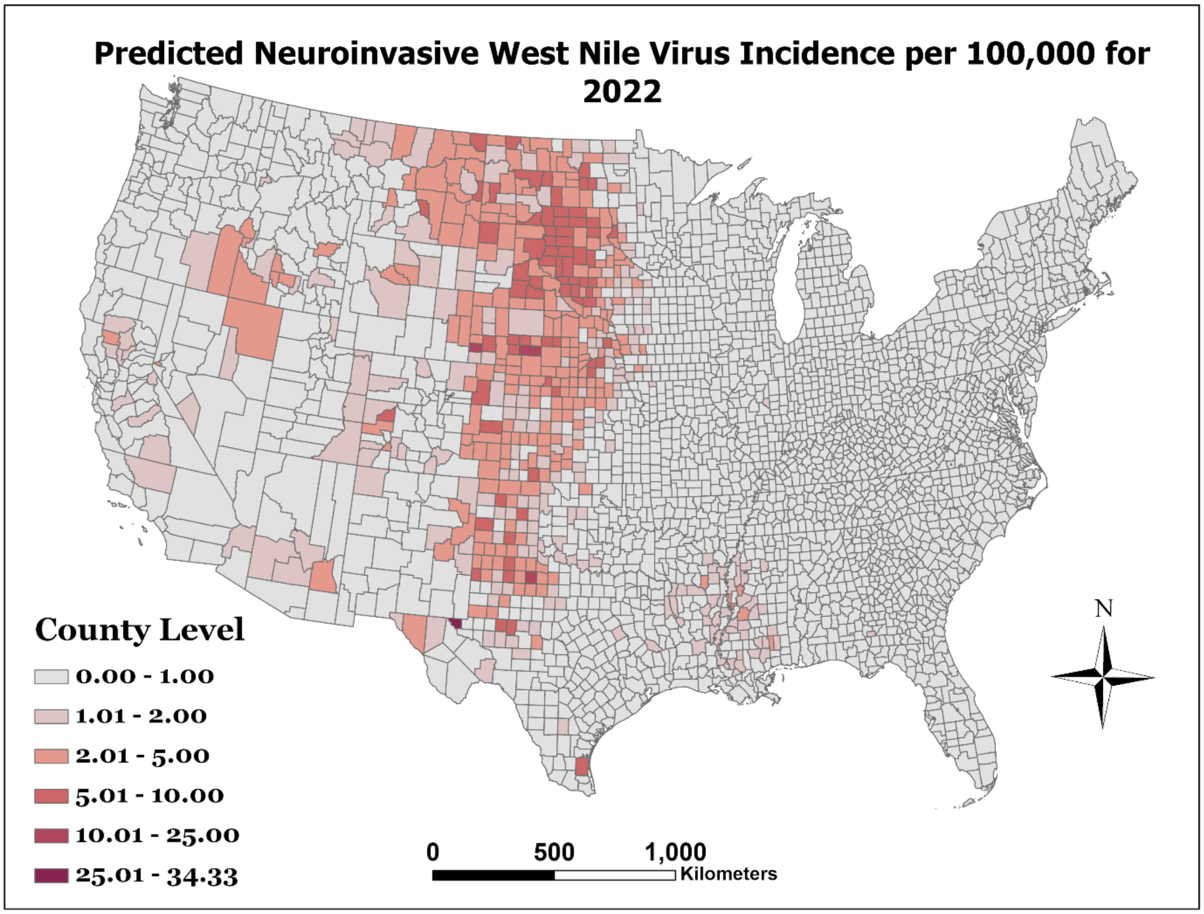

Web Figure 10 depicts the observed WNND counts plotted against the predicted counts from the final model for 2021.

Web Figure 10: Scatterplot of the Observed WNND Counts (X-Axis) versus the Predicted WNND Counts (Y-Axis) for 2021.

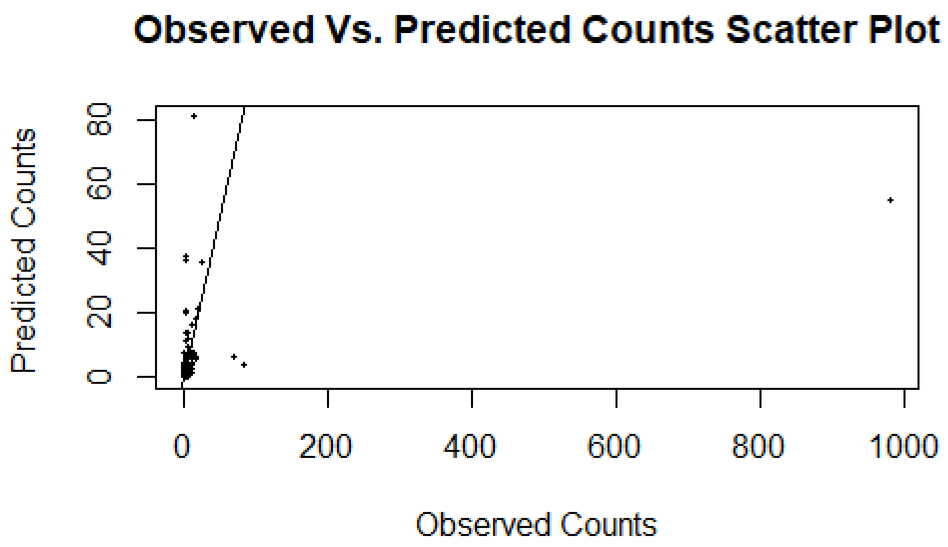

Web Appendix D: Available Data

Information on yearly neuroinvasive West Nile virus trends and count data are publicly available on ArboNET at: https://www.cdc.gov/westnile/statsmaps/historic-data.html. The covariate data and code used for this project is available at https://github.com/maggie-mccarter/WNND_INLA.
